# Meningitis Dipstick Rapid Test: Evaluating Diagnostic Performance during an Urban *Neisseria meningitidis* Serogroup A Outbreak, Burkina Faso, 2007

**DOI:** 10.1371/journal.pone.0011086

**Published:** 2010-06-11

**Authors:** Angela M. C. Rose, Judith E. Mueller, Sibylle Gerstl, Berthe-Marie Njanpop-Lafourcade, Anne-Laure Page, Pierre Nicolas, Ramata Ouédraogo Traoré, Dominique A. Caugant, Philippe J. Guerin

**Affiliations:** 1 Epicentre, Paris, France; 2 Chronic Disease Research Centre, University of the West Indies, Barbados, West Indies; 3 Agence de Médecine Préventive, Paris, France; 4 Institut de Médecine Tropicale du Service de Santé des Armées (IMTSSA), World Health Organization Collaborating Centre for Reference and Research on Meningococci, Marseille, France; 5 Laboratoire de Biologie, Centre Hospitalier Universitaire Pédiatrique Charles de Gaulle, Ouagadougou, Burkina Faso; 6 World Health Organization Collaborating Centre for Reference and Research on Meningococci, Norwegian Institute of Public Health, Oslo, Norway; 7 Institute of General Practice and Community Medicine, University of Oslo, Norway; Norwegian Knowledge Centre for the Health Services, University of Oslo, Norway

## Abstract

Meningococcal meningitis outbreaks occur every year during the dry season in the “meningitis belt” of sub-Saharan Africa. Identification of the causative strain is crucial before launching mass vaccination campaigns, to assure use of the correct vaccine. Rapid agglutination (latex) tests are most commonly available in district-level laboratories at the beginning of the epidemic season; limitations include a short shelf-life and the need for refrigeration and good technical skills. Recently, a new dipstick rapid diagnostic test (RDT) was developed to identify and differentiate disease caused by meningococcal serogroups A, W135, C and Y. We evaluated the diagnostic performance of this dipstick RDT during an urban outbreak of meningitis caused by *N. meningitidis* serogroup A in Ouagadougou, Burkina Faso; first against an in-country reference standard of culture and/or multiplex PCR; and second against culture and/or a highly sensitive nested PCR technique performed in Oslo, Norway. We included 267 patients with suspected acute bacterial meningitis. Using the in-country reference standard, 50 samples (19%) were positive. Dipstick RDT sensitivity (N = 265) was 70% (95%CI 55–82) and specificity 97% (95%CI 93–99). Using culture and/or nested PCR, 126/259 (49%) samples were positive; dipstick RDT sensitivity (N = 257) was 32% (95%CI 24–41), and specificity was 99% (95%CI 95–100). We found dipstick RDT sensitivity lower than values reported from (i) assessments under ideal laboratory conditions (>90%), and (ii) a prior field evaluation in Niger [89% (95%CI 80–95)]. Specificity, however, was similar to (i), and higher than (ii) [62% (95%CI 48–75)]. At this stage in development, therefore, other tests (e.g., latex) might be preferred for use in peripheral health centres. We highlight the value of field evaluations for new diagnostic tests, and note relatively low sensitivity of a reference standard using multiplex vs. nested PCR. Although the former is the current standard for bacterial meningitis surveillance in the meningitis belt, nested PCR performed in a certified laboratory should be used as an absolute reference when evaluating new diagnostic tests.

## Introduction

Although most large meningitis epidemics in sub-Saharan Africa are still caused by *Neisseria meningitidis* serogroup A [1], recent meningococcal outbreaks caused by serogroups W135 [2], [3] and X [4], [5] underline the importance of serogroup-specific bacteriological surveillance and outbreak investigation. Several vaccines with varying serogroup composition are available, and it is therefore crucial to identify the causative serogroup before launching reactive mass vaccination campaigns. In addition, the expected introduction of an affordable conjugate A vaccine in meningitis belt countries makes meningococcal surveillance crucial even during inter-epidemic periods [6].

*N. meningitidis* serogroups can be identified using antisera on culture, by polymerase chain reaction (PCR), and by latex agglutination tests such as Pastorex® (Bio-Rad Laboratories, Inc., Marne-la-Coquette, France) on cerebrospinal fluid (CSF). Culture isolation plus strain serogrouping and PCR-based methods are generally considered as the reference standard for identification of *N. meningitidis* serogroups, since they are highly specific. Although they necessitate a well-equipped laboratory with well-trained, specialised technicians, neither of which is often available close to the outbreak location, CSF samples can be tested by PCR after transport at ambient temperature in simple dry tubes, which makes it useful for surveillance in remote areas of sub-Saharan Africa [7]. If correctly performed on fresh samples from untreated patients, culture and PCR have been shown to have comparable performance [8]. Culture-based confirmation, however, is limited by the low yield of this method, due to a high susceptibility to contamination, e.g. after specimen preservation or transportation in sub-optimal conditions, or early antibiotic treatment [9]. On the other hand, no standardised PCR method for *N. meningitidis* detection has yet been established for use in European or African reference laboratories [9]. Some highly sensitive methods, such as nested PCR, used in supranational reference laboratories, are less adapted to field conditions due to the high probability of contamination, but are helpful as a reference standard.

Different producers have been developing meningococcal diagnostic tests better adapted for use in the field. Since 2002, Pastorex® test kits have been made available for the epidemic seasons in most countries in the meningitis belt, usually at district level laboratories. This test performs well but requires a minimum set of equipment (refrigerator, centrifuge and water bath), as the kit has to be stored at +4°C, and the CSF must be centrifuged and heated before performing the test [10], [11]. The Pastorex® test contains a mixture of anti-W135 and anti-Y reagents, and thus does not allow differentiation between these two serogroups. Recently, a new immuno-chromatography dipstick rapid diagnostic test (RDT), for the identification of *N. meningitidis* serogroups A, C, Y and W-135, was developed by the Centre de Recherche Médicale et Sanitaire (CERMES) in Niamey, Niger and the Pasteur Institute in Paris, France. This dipstick RDT principle is based on the detection of *N. meningitidis* serogroup capsular polysaccharide antigen through a one-step vertical-flow immunochromatography technique [12]. It does not require cold chain for storage, can be used directly on the CSF and can differentiate between the four *N. meningitidis* serogroups using an algorithm based on the results of two duplex dipsticks [12]. Under ideal laboratory conditions, the dipstick RDT sensitivity and specificity have been shown to be both between 93% and 100% [12]. However, during a previous *N. meningitidis* serogroup A outbreak in Niger in 2006, the dipstick RDT evaluated in peripheral health centres showed sensitivity and specificity of 89% and 62%, respectively [11]. This variation in results, especially the low specificity observed when the dipstick RDT was conducted in the field, warranted further evaluation.

Here we report the results of a field evaluation conducted in March-April 2007 during an *N. meningitidis* A outbreak in an urban setting in Ouagadougou, Burkina Faso. We evaluated the new dipstick RDT in comparison with an in-country reference standard of culture and/or multiplex PCR. In addition, we compared dipstick RDT results with those from culture combined with a highly sensitive nested PCR technique, conducted at the Meningococcal Reference Laboratory, Norwegian Institute for Public Health (NIPH) in Oslo, Norway.

## Methods

### Ethics statement

The study protocol was reviewed and approved by the ethical committee of the Ministry of Health in Ouagadougou, Burkina Faso, the Comité de Protection des Personnes, Ile de France XI, France and the ethics review board of Médecins Sans Frontières (MSF). Written consent was obtained from each participating patient or his/her legal guardian.

### Study population and procedures

The study was carried out in Ouagadougou, the capital of Burkina Faso, after declaration of an epidemic of *N. meningitidis* serogroup A in 2007. The study took place at three sites, two within Pissy district (Pissy Centre Médical avec Antenne Chirurgicale and Nagrin Centre de Santé et de Promotion Sociale (CSPS)), while the third site (CSPS Sector 15) fell in a different health district of Ouagadougou.

From 26 March (week 13) to 21April (week 16) 2007 inclusive, all patients aged over 2 months presenting at any of the three study sites with clinically suspected acute bacterial meningitis (see Appendix S1) [13] and from whom CSF was obtained, were included in the study. Previous experience has indicated that, during a meningitis outbreak, the proportion of positive CSF samples from patients fitting the case definition could be as high as 70% [14], [15]. For the sample size calculation, sensitivity was estimated at 89% (with a precision of ±5%) and specificity at 75% (precision ±10%). For an α error of 5%, a sample size of 280 was needed.

Treatment based on clinical symptoms (not results of the dipstick RDT being evaluated) was provided free of charge by MSF, according to the treatment protocol for meningococcal meningitis [13], and following national guidelines.

According to national guidelines, a lumbar puncture was performed by a trained nurse on each suspected meningitis patient. A sample of 3 ml CSF was collected into two sterile tubes. Tube A, containing 2 ml, was used for (1) macroscopic examination (appearance of CSF); (2) inoculation of CSF into trans-isolate (TI) medium for culture (1 ml); (3) performance of dipstick RDTs at study site (0.5 ml). Tube B, containing 1 ml CSF, was frozen for future PCR. Data collected for each suspected meningitis patient included the health centre name, patient's age, sex, place of origin, antibiotics/antimalarials already taken (where appropriate), whether meningitis vaccination was received before arrival to the health centre, symptoms and clinical signs.

### Tests conducted in the field in Burkina Faso

After examination of CSF appearance (cloudy, clear or bloody), dipstick RDTs were performed following the manufacturers' instructions by the treating nurse or physician at each of the three study sites. Briefly, seven drops of CSF were placed in each of two tubes. The two RDT dipsticks were placed in the tubes and left for 10 to 15 minutes before reading. Interpretation of the test was completed using the interpretation algorithm, which determines the serogroup based on the results of both dipsticks [12]. Results were classified as undefined if one or both of the lines of the RDT dipsticks were too faint to read.

### Tests conducted in the laboratory in Burkina Faso

CSF-inoculated TI media were transported weekly during the study to the Burkina Faso national reference laboratory, in the Charles de Gaulle Paediatric Hospital in Ouagadougou, where culture was performed on chocolate agar plates. Bacteria growing on these plates were identified using standard bacteriological techniques. Serogrouping of *N. meningitidis* was done using specific *N. meningitidis* anti-sera [16] (BD Difco™).

Frozen CSF samples were first sent to the PCR laboratory at Centre Muraz/Agence de Médecine Préventive (AMP) in Bobo-Dioulasso, Burkina Faso. After DNA extraction, an initial multiplex PCR was performed as described previously [17] using three pairs of primers for the identification of *N. meningitidis*, *Streptococcus pneumoniae* and *Haemophilus influenzae* (type b). If a sample was positive for *N. meningitidis*, the result was confirmed by a specific single PCR and a second multiplex PCR for genogrouping was performed using primers specific for the *siaD* and *mynB* genes [18].

All positive *N. meningitidis* isolates grown in culture were transported to the meningococcal unit at the Institut de Médecine Tropicale du Service de Santé des Armées (IMTSSA), the WHO Collaborating Centre for Reference and Research on Meningococci in Marseille, France, for confirmation and phenotype determination (serogroup, serotype and sub-type) by ELISA using monoclonal antibodies.

### Tests conducted in the supranational reference laboratory in Oslo

Remaining CSF specimens from B tubes were sent to the NIPH in Oslo. DNA was extracted using a QIAamp DNA mini kit (QiaGen, Oslo, Norway) according to the manufacturers' recommendations. Nested PCR was performed as described previously using primers specific for the *porA* gene [19]. If the nested *porA* PCR was positive, the PCR product was sequenced on both strands to determine the genosubtype of the infecting strain and the CSF was subjected to another PCR for genogrouping, using primers specific for the *siaD* and *mynB* genes [18].

### Analysis

Data were entered into EpiData 3.0 software (The EpiData Association, Odense, Denmark) and analysis was conducted using Stata 8.0 (Stata Corporation, College Station, Texas, USA).

As our analysis focused on the detection of the outbreak strain (*N. meningitidis* serogroup A), all results indicating other strains or serogroups were classified as negative.

We calculated sensitivity, specificity, positive and negative predictive values (PPV and NPV, respectively) with 95% confidence intervals (95%CI) for the dipstick RDT, using a reference standard of culture and/or multiplex PCR in which both of these tests were conducted at the laboratory in Burkina Faso. This combination reference standard has been used in prior evaluations of the RDT [11], [12]. Samples with positive results for either culture or PCR were defined as reference standard positive. Reference standard negatives were samples with both test results negative, or those with one test negative and the other either not done or with an undetermined result. Excluded from analysis were all samples with the dipstick RDT or both reference standard test results either missing or undetermined (samples with contaminated culture, inhibited PCR and undefined RDT results were classified as having undetermined results). The same performance indicators were calculated using a second reference standard, in which in-country culture was combined with nested PCR conducted at the NIPH in Oslo.

## Results

**Figure 1:**
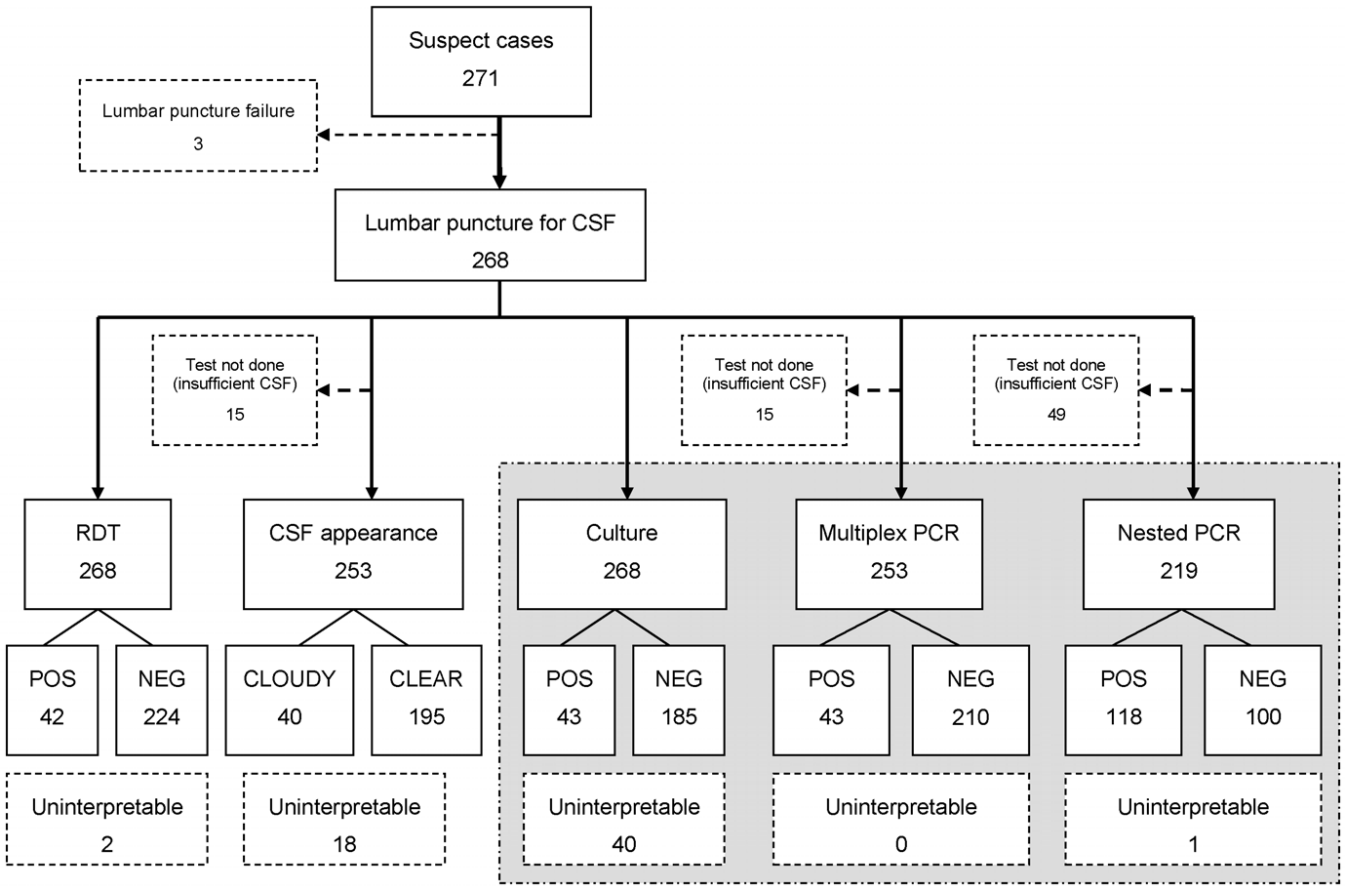
Schematic of all rapid and confirmatory diagnostic tests for *Neisseria meningitidis* conducted on cerebrospinal fluid (CSF) samples, showing results obtained for serogroup A. Shaded section shows confirmatory tests (‘reference standard’). (Note: RDT = dipstick rapid diagnostic test; ‘Uninterpretable’ for CSF appearance = bloody CSF, for culture = contaminated, for PCR = inhibited.)

A total of 268 patients fulfilled the inclusion criteria (Figure 1). Among 228 valid culture results, 43 (19%) were positive for *N. meningitidis* A and three (1%) were positive for *Streptococcus* spp. Among 253 valid in-country multiplex PCR results, 43 (17%) were positive for *N. meningitidis* A, and one (<1%) was positive for *S. pneumoniae*. Among 218 valid nested PCR results, 118 (54%) were positive for *N. meningitidis* A, and one (<1%) was positive for *N. meningitidis* serogroup X. Finally, among 266 valid dipstick RDT results, 42 (16%) were positive for *N. meningitidis* A, and two (<1%) were positive for *N. meningitidis* serogroup W135. Of the 122 samples on which genogrouping was performed, 56 were positive (46%). Of these, 55 were serogroup A and one was serogroup X (the remaining samples were positive on nested *porA* PCR only).

**Figure 2:**
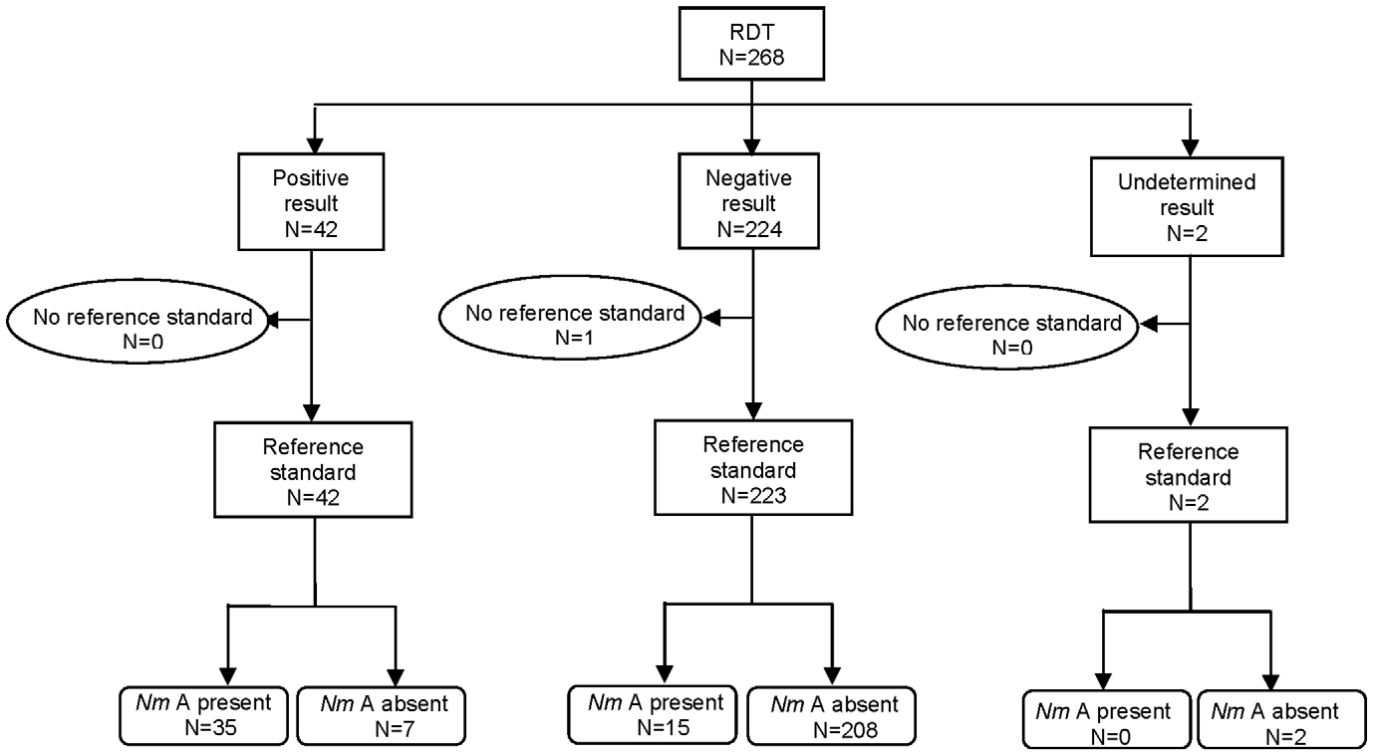
Flow diagram showing diagnostic performance of the dipstick rapid diagnostic test (RDT) against a reference standard of in-country culture and/or multiplex PCR conducted in Burkina Faso. (Note: “No reference standard” indicates those samples for which the reference standard result was undetermined or where there was not enough CSF remaining to conduct PCR.)

There were 265 CSF samples with either a positive or negative result for both the dipstick RDT and the in-country reference standard (Figure 2), from which we found dipstick RDT sensitivity to be 70% (95%CI 55–82), specificity 97% (95%CI 93–100), PPV 83% (95%CI 69–93) and NPV 93% (95%CI 89–96) (Table 1).

**Table 1:** Sensitivity, specificity, positive and negative predictive values (PPV and NPV, respectively) for the dipstick RDT* against a reference standard of culture and/or (a) multiplex PCR and (b) nested PCR.

	(a) Dipstick RDT vs culture and/or multiplex PCR (N = 265)	(b) Dipstick RDT vs culture and/or nested PCR (N = 257)
**Sensitivity % (95%CI)**	70 (55.4–82.1)	32 (23.9–40.9)
**Specificity % (95%CI)**	97 (93.4–98.7)	99 (94.6–99.8)
**PPV % (95%CI)**	83 (68.6–93.0)	95 (83.8–99.4)
**NPV % (95%CI)**	93 (89.1–96.2)	61 (53.6–67.0)
**Prevalence**	19 (14–24)	49 (42–55)

*RDT: Rapid diagnostic test.

Of the 50 patients with CSF samples positive by the in-country reference standard and having information on all three classical clinical signs for meningitis (fever, headache and stiff neck), 18 (36%) had all three. In contrast, only 40 of the 216 (19%) patients with negative samples by this reference standard had all three clinical signs present together (χ^2^ for difference between proportions = 7.3; p = 0.007; Table 2). Cloudy CSF was observed in 31/45 (69%) patients whose samples had been confirmed by this reference standard, vs 9/204 (4%) of patients for whom this reference standard was negative (χ^2^ = 113.7; p<0.0001; Table 2).

**Table 2:** Clinical signs and appearance of cerebrospinal fluid (CSF) samples by reference standard result.

	Cloudy CSF appearance (%)	Number with any one clinical sign* (%)	Number with all 3 clinical signs* (%)
**Samples positive by culture and/or multiplex PCR (N = 50)**	31/45 (69)	50/50 (100)	18/50 (36)
**Samples negative by culture and/or multiplex PCR (N = 217)**	9/190 (5)	207/216 (96)	40/216 (19)
**Samples positive by culture and/or nested PCR (N = 126)**	33/118 (28)	123/125 (98)	33/123 (26)
**Samples negative by culture and/or nested PCR (N = 133)**	7/109 (6)	127/133 (95)	24/133 (18)

* ‘Clinical signs’: the three classic clinical signs for meningitis (fever, headache and stiff neck).

**Figure 3:**
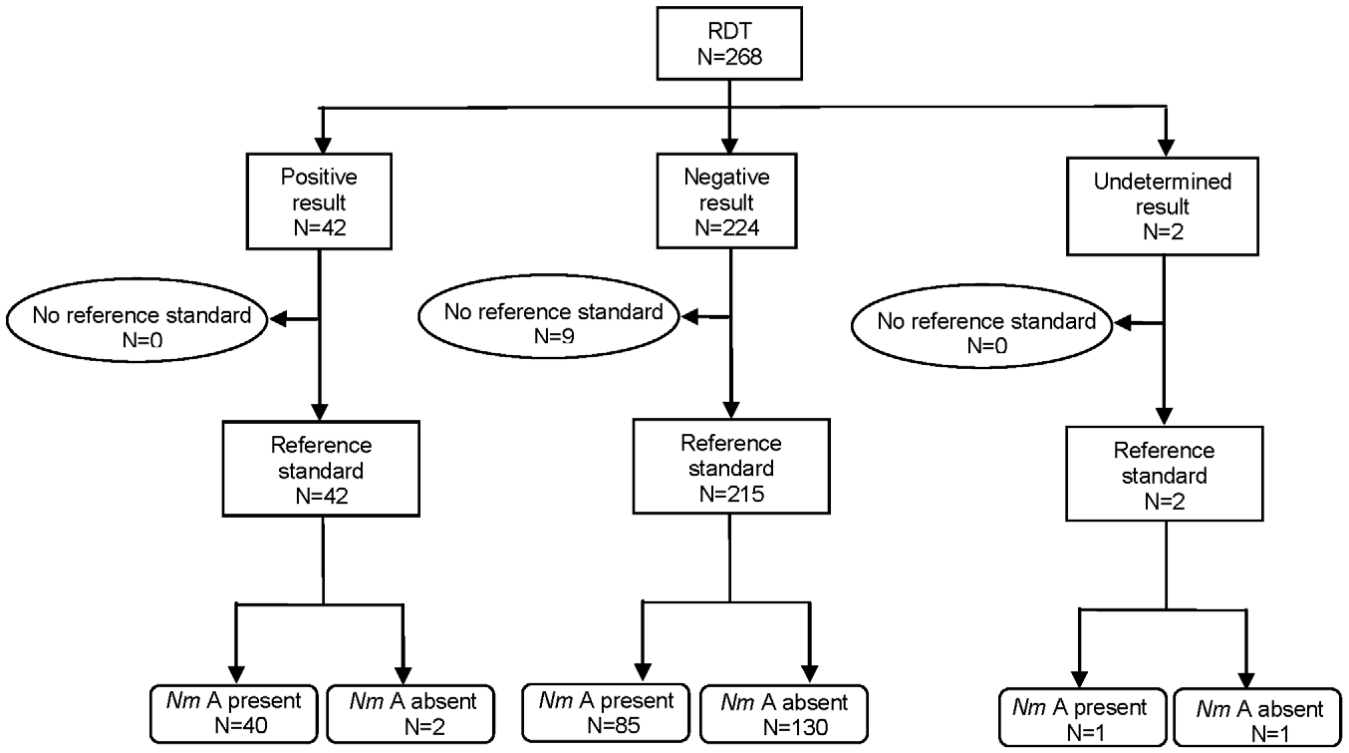
Flow diagram showing diagnostic performance of the dipstick rapid diagnostic test (RDT) against a reference standard of in-country culture and/or nested PCR conducted in Oslo. (Note: “No reference standard” indicates those samples for which the reference standard result was undetermined or where there was not enough CSF remaining to conduct PCR.)

In the comparison with a reference standard of culture and/or nested PCR (Figure 3), there were 257 CSF samples with a clear positive or negative result for both tests. We found dipstick RDT sensitivity to be 32% (95%CI 24–41), specificity 99% (95%CI 95–100), PPV 95% (95%CI 84–99) and NPV 61% (95%CI 54–67) (Table 1).

Among patients with positive or negative CSF samples by this reference standard, 33/125 (26%) and 24/133 (18%), respectively, had all three classical meningitis signs (χ^2^ = 2.6; p = 0.11). The CSF was cloudy in 33/118 (28%) positive samples vs 7/109 (6%) of negative samples (χ^2^ = 18.1; p<0.0001; Table 2).

Of the 268 patients included in the study, 31 (12%) had taken an antibiotic prior to arrival at the study site, and 190 (71%) reported having had a meningitis vaccination. Of the 190 previously vaccinated patients, 26/178 (15%) were positive by the reference standard using culture and/or multiplex PCR, while 82/182 (45%) previously vaccinated patients were positive by the reference standard which incorporated nested PCR. Vaccination cards were not observed, so these data were obtained by verbal confirmation only.

## Discussion

Using an in-country reference standard of culture and/or multiplex PCR, we show that dipstick RDT sensitivity at 70% (95%CI 55–82) was lower and specificity at 97% (95%CI 93–99) was substantially higher than found in an earlier dipstick RDT field evaluation using the same reference standard [89% (95%CI 80–95) and 62% (95%CI 48–75) respectively] [11]. The dipstick RDT sensitivity found in this study was also lower than reported from prior evaluations conducted using ideal laboratory conditions (89%; 95%CI 84–93 [20] and 94%; 95%CI 92–96 [12]). Specificity, however, was comparable to that found by these studies [94% (95%CI 92–96) [20] and 97% (95%CI 94–99) [12]].

Variation in dipstick RDT results may be explained by differences in the test quality, which could be due either to batch-to-batch performance variations or poor stability of the test. The latter should be considered especially as dipstick RDT kits were stored at room temperature, which reached 40°C during the study. Such variations have been largely documented for other rapid diagnosis tests, e.g. the malaria RDT using similar mechanisms [21], [22]. Further development of the dipstick RDT should ensure better and more reproducible results, although the mass production of this test in the future is not yet assured [23]. Importantly, these varying results (especially the poor sensitivity shown in the current study) indicate that this dipstick RDT should not be used at bedside for individual patient diagnosis. Conditional on sufficient batch-to-batch stability, this RDT may be useful to declare an outbreak, and to guide vaccination choice.

The variations in performance described here suggest that other rapid tests, such as the latex agglutination tests, are preferable for use at a peripheral laboratory level, once the necessary equipment is available. The latter is important, as earlier research emphasises that the latex agglutination tests must be used according to manufacturers' recommendations in order to achieve optimum sensitivity, requiring a certain level of human resources and infrastructure [11].

Although culture is known to have a low sensitivity, especially in cases where antibiotics have been administered to patients or if the sample collection and/or transportation are sub-optimal [24], [25], this technique remains indispensable to determine the antibiotic sensitivity profile and genetically characterize the outbreak strain. PCR is of added value due to its greater yield of valid results in remote areas, and its capacity to detect and serogroup other agents of bacterial meningitis, namely pneumococci, *Haemophilus influenzae* b and *N. meningitidis* serogroup X [8], [26]. PCR is generally considered to be more sensitive than culture [23], [27], since it can detect DNA of few dead or alive bacteria. However, previous studies in sub-Saharan Africa have shown comparable sensitivity and specificity of culture and PCR if performed on fresh CSF from untreated patients [8]. This study, therefore, like previous evaluations of rapid tests for *N. meningitidis* [11], [12], [15], used a reference standard which combined the locally available techniques of culture and mutliplex PCR. A highly sensitive nested PCR technique was added (also in combination with in-country culture) as an absolute reference. In a nested PCR, the initial PCR reaction is performed on the extract and the product of this first reaction is then used as a template for a second PCR reaction. While a reference standard including this technique was thus expected to have a better sensitivity than one incorporating multiplex PCR (which involved two multiplex and one specific PCR step), the magnitude of this difference was surprising. As the laboratory performing the multiplex PCR in this study was a high-quality national reference laboratory, this difference could be even higher if it had been performed in regional or district laboratories. Comparative analysis of the two PCR techniques (data not shown) revealed that of 118 samples positive for *N. Meningitidis* serogroup A by the more sensitive nested PCR technique, only 37 (31%) were positive by multiplex PCR (there were no additional samples positive by multiplex PCR, i.e. all samples negative by the nested technique were also negative by multiplex). Incorporating culture with the PCR results gave a similar result (48 culture-with-multiplex PCR out of 126 culture-with-nested PCR positive; 38%).

Although multiplex PCR is the current standard for bacterial meningitis surveillance in the meningitis belt, the more sensitive nested PCR technique would provide more accurate information for evaluation of a new diagnostic test. However, the greater risk of contamination during the nested PCR is much higher, so this technique may not be feasible as part of an in-country reference standard, although samples could be transported to a supranational reference laboratory certified for this procedure, as was done in our study. An alternative in-country method could be to use the *PorA* gene directly to indicate presence of *N. meningitidis*, followed by genogrouping (but should initially be compared with multiplex PCR).

A comparison study of PCR methods between several European laboratories has shown that the primers used for PCR identification of *N. meningitidis* give equivalent results [9]. However, this comparison study also demonstrated that results vary between laboratories, with one laboratory having a sensitivity of only 55% compared with the consensus results of all laboratories [9]. This underlines the importance of the type of PCR technique used for the diagnosis of bacterial meningitis, and the potential for variation between laboratories.

Apart from these technical considerations, the choice of laboratory method ultimately depends on how the results are being used; these may be different if used for (a) clinical diagnosis at an individual level (bedside), (b) surveillance to detect outbreaks and to guide vaccine choice, (c) estimating burden of disease during or outside epidemics, (d) surveillance to describe the etiological range of bacterial meningitis, or (e) surveillance to evaluate vaccine impact and strain replacement. This evaluation shows the value of the dipstick RDT for use in (b).

The dipstick RDT is clearly easier to use and to store, and requires less training for its users, than any of the prior rapid tests (such as Pastorex® or Slidex®) or the two other tests used in-country for the reference standard, culture and multiplex PCR. This advantage for outbreak investigation in remote settings should be carefully weighed against limitations in performance identified in the present study. To limit potential harm by inappropriate treatment decision following a negative dipstick RDT result, it may be necessary to restrict its use to staff not involved in routine patient care. Further evaluation of the field performance of the dipstick RDT in identifying other serogroups is needed, in particular serogroup W135 (for which a different vaccine is required). In this analysis, we treated two dipstick RDT results positive for *N. meningitis* W135, as negative (i.e. negative for the outbreak strain). Interestingly, one of these samples was positive for *N. meningitidis* A by both PCR techniques, while the other sample was negative by multiplex PCR (there was insufficient CSF for nested PCR to be performed). This highlights the need to investigate the dipstick RDT further for potentially misleading cross-reactivities.

Finally, although results from evaluations performed in well-resourced, air-conditioned laboratories with fully trained personnel may well be excellent indicators of a new test's theoretical optimal performance, they cannot and should not be assumed to apply equally to the field without thorough ‘operational’ testing, especially when they are to be used in the hot, dry, dusty areas with limited resources such as the African meningitis belt. Our studies illustrate that there is no substitute for ‘real’ field testing in diagnostics, and prove the value of operational research.

## Supporting Information

Appendix S1 Case definition for suspected acute bacterial meningitis patients used during the study [13].
(0.03 MB DOC)
