# Supplementary material for: Meningitis Dipstick Rapid Test: Evaluating Diagnostic Performance during an Urban Neisseria meningitidis Serogroup A Outbreak, Burkina Faso, 2007
Source: PLoS One. 2010 Jun 11;5(6):e11086. doi: 10.1371/journal.pone.0011086 (PMC2884039; doi:10.1371/journal.pone.0011086)
Supplement: Appendix S1 — Case definition for suspected acute bacterial meningitis patients used during the study [13]. (0.03 MB DOC) [file pone.0011086.s001.doc]

# Appendix S1. Case definition for suspected acute bacterial meningitis patients used during the study.[13]

(a) Children over 12 months, and adults:

Sudden high fever (measured on arrival to the health post  ≥ 38.5°C) associated with at least one of the following signs:

- Stiff neck
- Petechial or purpuric rash

(b) Infants from 2 to 12 months:

Sudden high fever (as above) associated with at least one of the following signs:

- Bulging fontanelle
- Petechial or purpuric rash

(*Note*: Infants under 2 months old were excluded from the study.)
